# French Adaptation and Validation of the International Outcome Inventory on Hearing Aids (IOI-HA) Questionnaire

**DOI:** 10.3390/audiolres15040097

**Published:** 2025-08-06

**Authors:** Maria-Pia Tuset, Mary Daval, Daniel Levy, Denis Ayache, Stéphane Gargula

**Affiliations:** Department of Otolaryngology, Fondation Adolphe de Rothschild Hospital, 29 rue Manin, 75019 Paris, France

**Keywords:** PROMs, questionnaire, hearing aid, hearing loss, psychometric analysis

## Abstract

**Objective:** Hearing rehabilitation using hearing aids keeps increasing in the general population. Patient-related outcome measures are essential to evaluate benefits. Although the IOI-HA is routinely used in France, its translated version from 2002 has never been validated. This study aimed to assess the psychometric properties of the French version of the IOI-HA questionnaire. **Design:** Controlled, prospective, monocentric study performed between February 2024 and January 2025. The forward–backward technique was used for translation of the questionnaire. **Study Sample:** 100 patients fitted with hearing aids completed the questionnaire. Thirty-five patients were retested 15 days after first completion. **Results:** Internal consistency, assessed by Cronbach’s alpha, was 0.863. Mean IOI-HA item scores ranged from 3.3 to 4.57. All seven items had a high degree of consistency with the total score, except for item Q1 which had a moderate score (0.45). Cronbach’s alpha after item deletion confirmed internal consistency. Intra-class correlation coefficients ranged from 0.622 (Q7) to 0.767 (Q5) and were all statistically significant (*p* < 0.001), revealing high reliability over time. No significant correlation was found between item scores and age, unilateral or bilateral hearing aid use or accompanying symptoms (tinnitus, dizziness). **Conclusions:** The French translation of the IOI-HA questionnaire, published in 2002, is a valid and reliable questionnaire evaluating hearing aid satisfaction. This validated questionnaire can now be used in daily clinical practice.

## 1. Introduction

Hearing loss represents one of the most prevalent and disabling conditions worldwide. According to the 2019 Global Burden of Disease (GBD) study, 1.57 billion individuals, around 20.3% of the global population, experience hearing loss. Among these are 403 million people experiencing moderate-to-complete impairment [1]. Europe reports a slightly lower prevalence of 16%, while in France, national estimates range from 11.1% based on GBD models to 24% according to more recent cohort studies [2,3].

Despite the availability of hearing rehabilitation technologies such as hearing aids, a large proportion of affected individuals remain untreated. In Europe, it is estimated that 77% of adults with disabling hearing loss do not use hearing aids. More specifically in France, an estimated 30% to 35% utilize hearing aids, equating to about 1.8 to 2.1 million users. This treatment gap has wide-reaching implications for communication, social participation, and quality of life. Effective rehabilitation requires not only appropriate device fitting but also structured follow-up and outcome assessment [3,4]. To address this need, patient-reported outcome measures (PROMs) have become central tools in evaluating hearing aid benefit and satisfaction [1,3,5].

Several questionnaires are available to assess hearing disability and hearing aid benefit, but only a few have been translated and validated in French [3,4]. To date, just three tools related to hearing performance have undergone full validation: the Speech, Spatial and Qualities of Hearing Scale (SSQ), the Hearing Handicap Inventory for the Elderly (HHIE), and the Satisfaction with Amplification in Daily Life (SADL). Others, such as the Hearing Handicap Inventory for Adults (HHIA) and alternate SSQ versions, have been translated but not psychometrically validated [3,4]. No questionnaire evaluating hearing aid outcomes has been validated in French. This limits both clinical use and the ability to compare results with international studies.

The International Outcome Inventory for Hearing Aids (IOI-HA) is a patient-related outcome measure (PROM) questionnaire designed to assess hearing aid outcomes. Its specific design aims to evaluate hearing aid efficacy across diverse populations [6]. Its seven items include the following topics: daily use (e.g., “On an average day, how many hours do you use your hearing aid(s)?”); perceived benefit (“How much have your hearing aid(s) helped you in your everyday life?”); residual activity limitations (“How much do your hearing difficulties still limit you in your daily life, even when using your hearing aid(s)?”); satisfaction (“How satisfied are you with your current hearing aid(s)?”); residual participation restrictions (“How much do your hearing difficulties still affect the things you can do with other people, even when using your hearing aid(s)?”); impact on others (“How much has your use of hearing aid(s) affected how others react to you?”); and overall quality of life (“Considering everything, do you think your quality of life has improved since you started using hearing aid(s)?”). Each item has five possible responses, ranging from the poorest (score = 1) to the most favorable outcome (score = 5). The original English validation showed that the items cluster into two factors. The first includes use, benefit, activity limitation, and satisfaction (items 1 to 4). The second includes participation, impact on others, and quality of life (items 5 to 7).

The IOI-HA is routinely used in France, and although a French translation was proposed in 2002 it has never been validated. As a result, it cannot be used reliably for research purposes or for comparing data across studies [7]. Researchers across the world have aimed to validate the psychometric properties of this questionnaire, proving its value as a PROM [8,9,10]. Direct translations without proper validation can lead to misinterpretations, compromising data integrity. Linguistic validation is essential to preserve the psychometric integrity of the instrument, particularly its internal consistency and construct validity.

Studies have described associations between hearing aid satisfaction and demographic or clinical factors. Properly fitted hearing aids that offer effective tinnitus masking have been shown to improve patient satisfaction, particularly when tinnitus-related distress decreases alongside auditory amplification [11]. Factors such as age, hearing loss severity and accompanying symptoms such as tinnitus and dizziness may impact on hearing aid fitting results and patient satisfaction [11,12,13]. Confirming or disproving these relationships would support the questionnaire’s robustness and cross-group comparability.

This study aims to validate the French version of the IOI-HA by assessing its internal consistency, test–retest reliability, and construct validity through exploratory factor analysis, within the French-speaking hearing-impaired population. The findings will provide clinicians and researchers with a reliable tool to assess hearing aid outcomes, facilitating improved patient care and contributing to the global applicability of the IOI-HA.

## 2. Materials and Methods

### 2.1. Design

A prospective, single-center study was conducted at the ENT outpatient clinic of the Fondation Adolphe de Rothschild Hospital in Paris, over a 12-month period between February 2024 and January 2025. The study was conducted as part of a structured program to evaluate hearing aid outcomes within routine clinical practice. Approval was obtained by the institutional review board with ID 2023-A01628-37. All patients provided written informed consent for participation and data use. Written permission was obtained from Robyn Cox and Genevieve Alexander, the authors of the original IOI-HA questionnaire.

The study was conducted in two phases: phase one consisted of the linguistic validation of the existing French version of the IOI-HA, and phase two consisted of psychometric evaluation and validation of the questionnaire in French.

### 2.2. Phase One: Linguistic Validation

Validation was performed using the French version of the IOI-HA published in 2002 [7]. This version was chosen because it is already widely used by audiologists and otologists in France. The questionnaire covers key aspects of hearing aid use, such as benefit and satisfaction. These themes are considered relevant across most hearing-impaired populations. Cultural adaptation challenges were limited. The questions address common issues in hearing rehabilitation, including device use, remaining difficulties, and daily impact.

To ensure consistency with international standards, a forward–backward translation was performed, following the 2017 ITC guidelines described by Hall et al. [9,10]. Two native French-speaking ENT specialists fluent in English completed independent forward translations. A consensus meeting followed, using a modified Delphi process. A third investigator moderated a live discussion. Agreement of at least 80% was required to finalize wording. The reconciled version became the forward French draft. A dual back-translation was then performed by two native English translators (one medical, one non-medical), blinded to the original text and to each other’s work. The two back-translations were compared with the original IOI-HA. Only minor wording differences were found and resolved by consensus.

**Figure 1:**
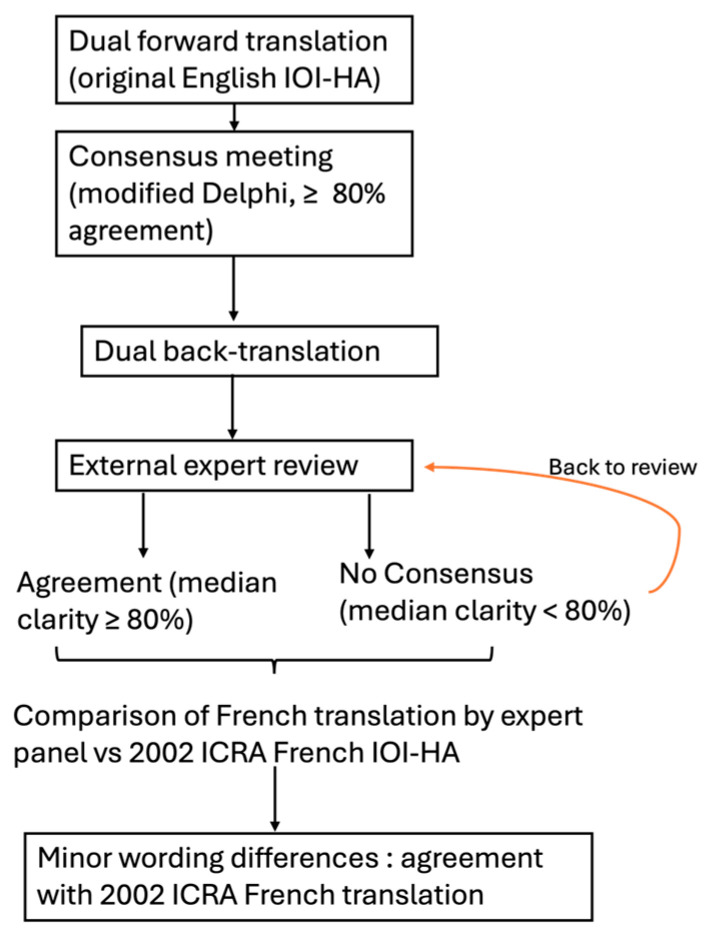
Flowchart summarizing the steps taken to compare the French translation of the IOI –HA performed by our expert panel to the ICRA published French translation of the IOI-HA.

Because the 2002 French version is commonly used in clinics, the harmonized draft was reviewed alongside the published version. A panel of experts compared the two versions. No semantic or syntactic discrepancies of practical consequence were identified, allowing us to proceed with psychometric evaluation of the available French version published in 2002. Eight additional French-speaking otolaryngologists, not involved in the earlier steps, rated each item for clarity and cultural appropriateness on a 4-point scale (1 = unclear, 4 = very clear). Figure 1 summarizes the methodology of the translation.

### 2.3. Phase Two: Psychometric Evaluation

#### 2.3.1. Participants

The psychometric properties of the French translation of the IOI-HA questionnaire were evaluated through the analysis of responses collected from a cohort of 100 adult patients. All patients presented hearing loss and had been fitted with hearing aids for at least six months. Patient recruitment was conducted consecutively during routine specialized consultations within the otology clinic. All participants were required to meet the following inclusion criteria: age of 18 years or older, no objection to study participation, and adequate comprehension of written French, subjectively assessed by the investigating clinician. Patients were excluded if they presented any cognitive impairment likely to interfere with the reliable completion or interpretation of the questionnaire, or if they were deemed unable to adequately understand its content. All participants were first met by the clinician, who explained the study objectives and provided standardized instructions on how to complete the questionnaire. While awaiting their audiometric assessment, patients filled out the form in the waiting area. Upon returning for their post-test consultation, the clinician collected the completed questionnaires for subsequent data entry and analysis. In France, the hearing aid fitting process is performed outside the hospital setting, by licensed hearing aid dispensers (hearing instruments specialists). They are responsible for the initial device fitting, adjustments, and user instruction and fitting follow-up. This process is typically not supervised or documented by hospital-based ENT departments. Accordingly, no fitting data was collected for this cohort.

#### 2.3.2. Test–Retest Reliability Assessment

To assess temporal stability of the questionnaire, a subgroup of 35 participants was invited to complete the IOI-HA a second time. A retest was then performed 15 to 30 days after the initial administration. The retest questionnaire was sent digitally by email two weeks later. Patients were invited to complete it at home and return it by email. If needed, assistance was provided by phone to clarify the instructions or facilitate completion.

The retest was conducted without any changes to the participants’ hearing aids or audiological status.

### 2.4. Procedure

The COSMIN (COnsensus-based Standards for the selection of health Measurement INstruments) reporting guidelines for studies on measurement properties of patient-reported outcome measures were followed to report and analyze the psychometric evaluation [14]. Data was collected from each patient’s medical file (age, gender). As part of the standard otologic assessment, all participants received a detailed clinical examination including otoscopy. Audiometry testing was conducted in accordance with World Health Organization (WHO) guidelines [15]. Pure-tone average (PTA) was calculated as the average of the hearing thresholds at the 0.5, 1, 2 and 4 kHz frequencies, in air conduction audiometry. Hearing loss severity was performed according to the WHO grading system [15].

### 2.5. Statistical Analysis

Statistical analyses were conducted using Microsoft Excel (Microsoft Corp., Redmond, WA, USA) and GraphPad Prism version 10.2.0 (GraphPad Software, San Diego, CA, USA). Normally distributed variables were analyzed using Student’s *t*-test, while the Mann–Whitney test was applied for non-normally distributed data. Spearman’s rank correlation was used to assess the association between each item and the total IOI-HA score, with items showing a correlation coefficient below 0.3 considered for potential deletion due to limited contribution. Internal consistency was calculated using Cronbach’s alpha (95% confidence interval). The impact of each item was evaluated by recalculating Cronbach’s alpha after deletion of each item, to determine its impact on internal consistency. Prognostic factors influencing questionnaire responses were explored using linear regression, to evaluate the potential impact of age, hearing loss severity, and accompanying symptoms (tinnitus and dizziness) on the total IOI-HA score. An exploratory factor analysis was also conducted to explore the underlying structure of the questionnaire and identify potential subscales. Test-retest reliability was evaluated using intraclass correlation coefficient (ICC, two-way mixed effects model, absolute agreement). An ICC value above 0.75 indicated good reliability. A *p*-value < 0.05 was considered statistically significant. Exploratory factor analysis was performed, using the Kaiser–Meyer–Olkin (KMO) measure for sampling adequacy. Bartlett’s test of sphericity was used to assess factorability. Principal-Axis Factoring (PAF) was selected to model shared variance across items. We re-analyzed the IOI-HA items using a polychoric correlation matrix and the WLSMV estimator in R (lavaan 0.6-17).

## 3. Results

### 3.1. Linguistic Validation

Minor wording adjustments were required after the back-translation comparison. Each item was judged independently, and consensus was reached that all seven items were applicable without cultural modification. While items related to benefit and satisfaction were deemed universally applicable, the panel also confirmed the relevance of more context-sensitive items such as residual participation restrictions and impact on others. Cultural adaptation challenges were considered minimal, and no items required rewording or exclusion. No conceptual changes were needed. The side-by-side review with the 2002 French version revealed no substantive differences, and the legacy wording was therefore retained for the validation study.

Expert panel ratings confirmed excellent clarity: the median score across the seven items was 4.0 (inter-quartile range 0). Because the predefined ≥ 3/4 threshold was satisfied for every item, no further revision loop was necessary in alignment with ITC Guideline 3.4, that allows expert evidence to substitute for lay testing when clarity is unequivocal.

### 3.2. Demographic Analysis

A total of 100 patients (62 women and 38 men), aged 64 ± 14 years [24–89, min–max], completed the questionnaire. Among them, 81% were bilateral hearing aid users. The average pure-tone audiometry (PTA) thresholds were 40 ± 19 dB HL [2–99] in the better ear and 54 ± 21 dB HL [32–120, min–max] in the worse ear. Global participant characteristics are detailed in Table 1. The exact duration of use was not systematically recorded. The majority were experienced users (56% had over 1 year hearing aid use, 15% under a year and data was missing in 29% of patients).

### 3.3. Global Psychometric Analysis

**Figure 2:**
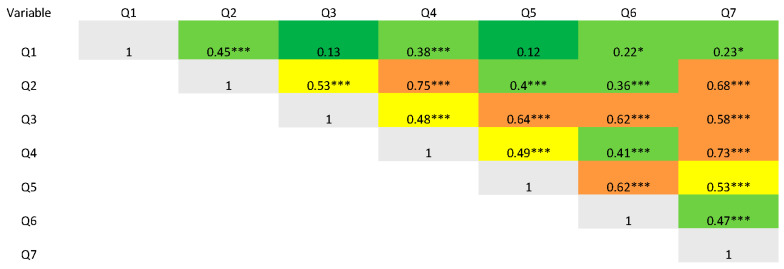
Heat map representing correlation coefficients of items with each other. * *p* < 0.05, *** *p* < 0.001. Correlations are calculated with Spearman’s rank correlation, red represents complete correlation, orange represents a ≥ moderate correlation, yellow represents a mild correlation, light green represents < mild correlation and dark green a lack of correlation.

The psychometric analysis of the IOI-HA items can be found in Table 2. All mean scores fell between 3.3 ± 1.1/5 (Q1, daily use) and 4.6 ± 0.7/5 (Q4, satisfaction). Internal consistency, evaluated with Cronbach’s alpha was 0.86 (95% CI [0.85, 0.91]). Item–total correlation was moderate to strong for all items. Items Q2 to Q7 had strong correlations ranging from 0.74 to 0.82, indicating a high degree of consistency with the total score. Q1 showed a lower ITC (0.45). Item deletion Cronbach’s alpha ranged between 0.86 and 0.90, confirming that no single item substantially detracted from internal consistency. Furthermore, Cronbach’s alpha remained stable across individual item deletions and closely matched the overall alpha coefficient, indicating strong internal consistency and robust reliability of the scale. The correlation of items between one another is presented in Figure 2.

### 3.4. Structural Analysis

Exploratory factor analysis (EFA) was conducted to examine the underlying structure of the IOI-HA questionnaire. Sampling adequacy was acceptable (KMO = 0.82), and Bartlett’s test confirmed the suitability of the data for factor analysis (χ^2^(21) = 362.52, *p* < 0.001). Both parallel analysis and the scree plot supported a one-factor solution. Principal-axis factoring yielded an eigenvalue of 4.10, accounting for 68% of the common variance. Factor loadings ranged from 0.68 to 0.85 (Table 3). Item 7 (residual activity limitation) showed the highest loading (λ = 0.85) and increased Cronbach’s alpha from 0.85 (Q2–Q6) to 0.88 (Q2–Q7), supporting its inclusion. Item 1 (daily use) had a lower loading (λ = 0.42) but was retained for its relevance. Commonalities ranged from 0.18 (Q1) to 0.71 (Q7).

Given the ordinal nature of the IOI-HA items, the EFA was repeated using a polychoric correlation matrix and the WLSMV estimator (lavaan, R 4.3.2). This model yielded a comparable solution, with a single latent factor (eigenvalue = 4.02) explaining 66% of the common variance (factor R^2^ = 0.66). Loadings for Q2–Q7 remained stable (0.66–0.83), and Q1 again showed a moderate loading of 0.42.

### 3.5. Reliability

Test–retest reliability, assessed with a two-way random-effects intraclass correlation, ICC (2,1), was 0.79 (95% CI [0.70, 0.86], *p* < 0.001). Item-level ICCs ranged from 0.62 (Q7) to 0.77 (Q5) and were all statistically significant (*p* < 0.001). The overall ICC was 0.79, indicating good reliability (Table 2). The first questionnaire was administered on-site and the retest by email. Paired *t*-tests showed no significant differences for any IOI-HA item or for the total score (all *p* ≥ 0.28; Table S1 in Supplementary Material). The absolute-agreement ICC remained good (ICC = 0.79), indicating that the change in administration mode did not impact outcomes.

### 3.6. Linear Regression Analysis

**Figure 3:**
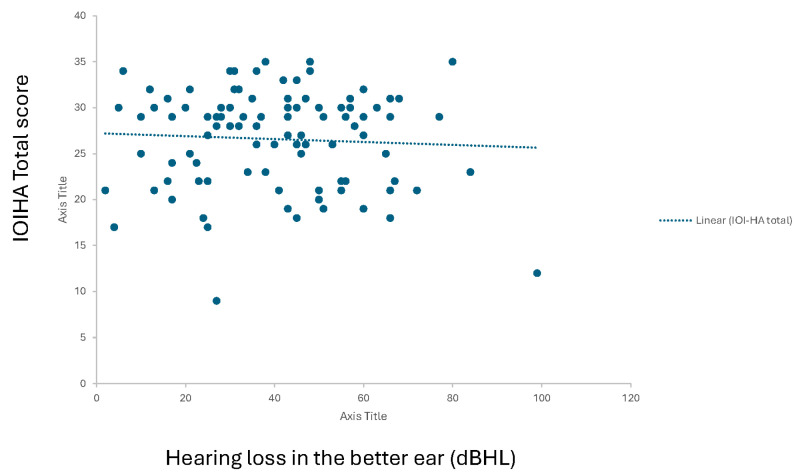
Linear regression curve representing hearing threshold correlation to IOI-HA total score (0 to 35).

Simple linear regressions showed no association between IOI-HA total score and age (univariate least-squares regression, β = 0.05, t(98) = 0.55, *p* = 0.59) or hearing thresholds in the better-ear PTA (β = −0.04, t(98) = −0.59, *p* = 0.56) or the worse-ear PTA (β = −0.12, t(98) = −1.61, *p* = 0.11). Linear regression plotting for hearing loss in the better ear is presented in Figure 3.

### 3.7. Exploratory Subgroup Comparisons

Severity of hearing loss, unilateral or bilateral hearing aid use, and the presence of coexisting symptoms (tinnitus, dizziness) had no impact on IOI-HA scores (Table 4).

To evaluate association of hearing and IOI-HA scores, we treated PTA as a continuous variable. No meaningful associations with IOI-HA outcomes were found (Table S2, Supplementary Material). Correlations ranged from −0.21 to 0.14 (all adjusted *p* > 0.007). Regression slopes were low (under 0.016 points per dB), explaining at most 4% of variance in any item. Independent-samples *t*-tests were performed for hearing aid fitting type (t(98) = 1.12, *p* = 0.27, d = 0.22), tinnitus (t(98) = 0.59, *p* = 0.56, d = 0.12) and dizziness (t(98) = 0.51, *p* = 0.61, d = 0.10).

## 4. Discussion

### 4.1. Psychometric Performance

The global score and individual items of the French IOI-HA demonstrated a strong discriminatory capacity for the questionnaire. Internal consistency was confirmed, with a Cronbach’s alpha of 0.86. Item-deletion effect on Cronbach’s alpha and item–total correlations (ITC) were calculated to assess individual item contribution to overall consistency. Results confirmed satisfactory consistency for all items. Intra-class correlation coefficients further supported test reliability over time, confirming temporal stability.

These results align with psychometric values of the English version validation by Cox et al. in 2002 [6]. In fact, similar means for each item were found, with elevated scores for each item (above 3.3) [6,16]. ITC was also comparable to psychometric values of the English version. In fact, the lowest score in our analysis was found for Q1, which was the second lowest score in the English version after Q5. This may suggest that Q1 is a subjective evaluation of patient motivation rather than a hearing aid outcome. Daily use can depend on habits, expectations, or lifestyle, not just the device’s benefit. This could explain its lower correlation with other items and supports prior findings identifying Q1 as a distinct construct [12,16]. The highest ITCs in both versions were obtained in Q4 and Q7. Cronbach’s alpha after item deletion was above 0.7 in both analyses, confirming high reliability of the test in the French translation also.

Parallel analysis supported a single dominant factor in our sample. In the psychometric analysis of the English version, a two-factor structure emerged [16]. Factor 1 was termed as “me and my hearing aids” and factor 2 as “me and the outside world”. Nevertheless, in our analysis, when a two-factor solution is forced, the items partition as in the original English questionnaire. This indicates two coherent content clusters embedded within the overall construct. The second eigenvalue (0.88), however, fell below the parallel-analysis threshold, so we interpret these clusters as sub-themes rather than independent dimensions. These results were confirmed in translations in other languages [10,16,17]. Item Q1, with weak inter-item correlations and a moderate factor loading of λ = 0.42, falls out of these categories. The discrepancy observed may be explained by its limited score variability. Most respondents scored this item at the high end of the scale (4.57/5), consistent with a ceiling effect. Similar findings have been reported in prior IOI-HA validations. This leads us to hypothesize that duration of daily use may reflect the patient’s need of amplification, rather than its satisfaction. Thus, we retained it for descriptive purposes. We also evaluated internal correlation of the French version, which was not evaluated in the English analysis, by performing a test–retest 15 days after first responses. Our analysis supports results of previous translations proving stability of responses over time [16,17].

### 4.2. Limitations and Future Directions

Limitations of our study include the fact that no control group was used for comparison since all patients had to be hearing aid users. Furthermore, we did not perform external validation of the questionnaire by comparing it to another validated questionnaire evaluating hearing aid satisfaction. Nonetheless, the methodology is in line with those adopted in other translated versions of the IOI-HA [6,8,10,17]. The sample size also limited the scope of multivariate analyses. In particular, the subgroup of patients with profound hearing loss was underrepresented. Including a larger number of these patients would have strengthened the analysis, especially since this group consistently showed the lowest IOI-HA total scores. However, this low score may not necessarily indicate poor hearing aid fitting, but rather reflect the limitations of hearing aids in addressing profound hearing loss. Patients in this category often require more advanced rehabilitation strategies, such as cochlear implantation. Therefore, their dissatisfaction with hearing aids may be more indicative of an inadequate treatment modality than of device-related issues. Therefore, we do not consider the lack of patients with profound hearing loss as a bias in our study, since they do not represent the target population of a questionnaire focused on hearing aid fitting. Another limitation is the change in administration format between test and retest session, with the latter performed by email. Mode of administration has been shown to influence responses in some hearing-related questionnaires [18,19]. Nevertheless, our results suggest that the French IOI-HA retained its reliability under these conditions. All mean differences were non-significant, and the test-retest ICC (0.79) indicated stable reproducibility across formats. Future studies should explore equivalence between digital and paper versions more systematically. In addition, the data concerning hearing aid fitting history was insufficient in our population. This prevented analysis of how fitting experience might influence outcome scores, particularly for items such as daily use. While our subgroup comparisons did not yield statistically significant differences, we acknowledge the limitations of our sampling framework. Most participants had mild to moderate hearing loss, and nearly all were current hearing aid users. These characteristics likely reflect a motivated clinical population with prior access to audiological care, which may bias results toward higher self-reported benefit and satisfaction. Selection bias could inflate score estimates and attenuate variability. Although these subgroup analyses are informative, definitive conclusions regarding normative scores, or intergroup differences cannot be drawn from our cohort. These analyses are best viewed as exploratory. Future studies using representative population sampling and stratified recruitment are needed to establish normative IOI-HA values for the French-speaking population and validate our subgroup comparisons.

This validation supports the use of the IOI-HA in clinical research in France and allows comparison of outcomes with international cohorts. It also opens the way for developing new PROMs that explore other aspects of hearing aid rehabilitation. The IOI-HA can be used as a reference tool and compared with other questionnaires, such as the COSI (Client-Oriented Scale of Improvement) [16,17], to strengthen future studies and validate other frequently used questionnaires. The psychometric properties of the French IOI-HA translation published in 2002 by Cox et al. had never been evaluated [7]. This study validates this French version and its use in daily practice to evaluate hearing aid users.

## 5. Conclusions

This study confirms that the French version of the IOI-HA is a valid and reliable tool to assess hearing aid outcomes in daily clinical practice. The questionnaire showed good internal consistency and test–retest reliability, with results in line with previous international validations. Given its simplicity and strong psychometric properties, the French IOI-HA can be confidently used for both clinical and research purposes in French-speaking populations.

## Figures and Tables

**Table 1:** Demographic characteristics of the population.

**Number**	**100**
Age (mean ± SD)	64 ± 14
Sex ratio (M/F)	38/62
Mean PTA (worst ear) (%)	54 ± 21
Mean PTA (better ear) (%)	40 ± 19
Hearing loss severity in better ear (dBHL)	
Not significant (16–25)	21%
Mild (26–39)	29%
Moderate (40–55)	25%
Moderately severe (56–70)	20%
Severe (71–90)	4%
Profound (>90)	1%
Type of deafness (%)	
Sensorineural	70%
Conductive	3%
Mixed	27%
Hearing aid fitting (%)	
Unilateral	19%
Bilateral	81%
Dizziness (%)	57%
Tinnitus (%)	66%

**Table 2:** Psychometric analysis of the IOI-HA items. Item–Total correlation (ITC), Cronbach with Item Withdrawal, and Internal Correlation Coefficient (ICC) are presented.

Item	Wording (Abridged)	Mean	Item–Total Correlation (ITC)	Cronbach with Item Withdrawal	Mean Difference	*p*-Value	Internal Correlation Coefficient (ICC)	*p*-Value
Q1	Hours of daily use	4.57	0.452	0.900	−0.0556	0.5347	0.7604	1.16 × 10^−7^
Q2	Benefit in listening situations	3.89	0.794	0.862	0.0278	0.8308	0.7180	1.20 × 10^−6^
Q3	Residual activity limitations	3.30	0.790	0.863	−0.0833	0.4746	0.6972	3.27 × 10^−6^
Q4	Satisfaction with the device	4.02	0.813	0.859	0.0278	0.8308	0.7268	7.64 × 10^−7^
Q5	Residual participation restrictions	3.34	0.759	0.870	−0.1667	0.1602	0.7677	7.38 × 10^−8^
Q6	Impact on others	3.71	0.739	0.874	−0.0833	0.5710	0.6470	2.67 × 10^−5^
Q7	Quality of life	3.76	0.816	0.859	−0.0278	0.8382	0.6226	6.51 × 10^−5^
Total		26,59	0.645	-	−0.361	0.5062	0.7968	1.0225 × 10^−8^

**Table 3:** Factor loadings * and item diagnostics for IOI-HA questionnaire.

Item	Loading (λ)	Communality	Item–Total r
Q1	0.42	0.18	0.32
Q2	0.82	0.67	0.71
Q3	0.79	0.63	0.69
Q4	0.83	0.7	0.73
Q5	0.75	0.56	0.64
Q6	0.68	0.51	0.61
Q7	0.85	0.71	0.74

* Extraction = principal-axis factoring (one-factor), unrotated. Loadings ≥ 0.40 are considered salient.

**Table 4:** Mean IOI-HA score depending on type of hearing aid fitting (unilateral or bilateral) and other symptoms (dizziness and tinnitus).

Variable	Mean Score	*p*-Value
Unilateral HA	26.42	0.22
Bilateral HA	26.63	
Tinnitus	25.95	0.56
Dizziness	25.91	0.61

## Data Availability

The data presented in this study is available on request from the corresponding author due to patient confidentiality reasons.

## Supplementary Materials

The following supporting information can be downloaded at: https://www.mdpi.com/article/10.3390/audiolres15040097/s1, Table S1: presenting item by item comparisons of data between test and retest (n = 36); Table S2: presenting correlation analysis between each item and hearing loss severity, considering PTA as a continuous variable (n = 100).
