# Supplementary material for: French Adaptation and Validation of the International Outcome Inventory on Hearing Aids (IOI-HA) Questionnaire
Source: Audiol Res. 2025 Aug 6;15(4):97. doi: 10.3390/audiolres15040097 (PMC12382775; doi:10.3390/audiolres15040097)
Supplement: Supplementary file 1 [file audiolres-15-00097-s001.zip › audiolres-3694057-supplementary.pdf]

Supplementary material :

Table S1 presenting item by item comparisons of data between test and retest (n=36)

| Item | Mean Test | SD Test | Mean Retest | SD Retest | Difference(Δ) | t | p |
|---|---|---|---|---|---|---|---|
| Q1 | 4,67 | 0,72 | 4,58 | 0,69 | -0,08 | -0,48 | 0,638 |
| Q2 | 3,81 | 0,92 | 3,78 | 0,99 | -0,03 | -0,12 | 0,906 |
| Q3 | 3,25 | 0,97 | 3,22 | 0,83 | -0,03 | -0,12 | 0,903 |
| Q4 | 4,14 | 1,02 | 3,89 | 1,01 | -0,25 | -1,09 | 0,285 |
| Q5 | 3,22 | 1,05 | 3,36 | 0,96 | 0,14 | 0,6 | 0,555 |
| Q6 | 3,69 | 1,06 | 3,58 | 1,05 | -0,11 | -0,43 | 0,672 |
| Q7 | 3,69 | 1,04 | 3,64 | 0,93 | -0,06 | -0,23 | 0,817 |

Table S2 presenting correlation analysis between each item and hearing loss severity, considering PTA as a continuous variable (n=100).

| Item | r | p (corr) | slope | $R^2$ |
|---|---|---|---|---|
| Q1 | 0.14 | 0.165 | 0.006 | 0.02 |
| Q2 | 0.085 | 0.4 | 0.004 | 0.007 |
| Q3 | -0.139 | 0.169 | -0.008 | 0.019 |
| Q4 | 0.084 | 0.409 | 0.004 | 0.007 |
| Q5 | -0.2 | 0.046 | -0.012 | 0.04 |
| Q6 | -0.214 | 0.033 | -0.013 | 0.046 |
| Q7 | 0.028 | 0.786 | 0.001 | 0.001 |
| Total | -0.058 | 0.565 | -0.016 | 0.003 |
